# Crossing Borders: SRH Challenges Among Immigrant and Minority Adolescents

**DOI:** 10.3390/ijerph22071101

**Published:** 2025-07-12

**Authors:** Patience Castleton, Ahmed Shabbir Chaudhry, Negin Damabi, Salima Meherali, Zohra S. Lassi

**Affiliations:** 1 Robinson Research Institute, University of Adelaide, Adelaide, SA 5005, Australia; 2 School of Public Health, Faculty of Health and Medical Sciences, University of Adelaide, Adelaide, SA 5000, Australia; 3 Faculty of Nursing, University of Alberta, Edmonton, AB T6G 1C9, Canada

**Keywords:** sexual and reproductive health, adolescent, migrants, minority population

## Abstract

The adolescent years are pivotal in reproductive and sexual development and maturation, yet the experience of migration can severely disrupt this period, inhibiting young immigrants’ knowledge, access, and engagement with sexual and reproductive health (SRH) services. Further, young immigrants and minority populations often face persistent intersectional barriers, including language difficulties, cultural stigma, and systemic exclusion, that result in adverse SRH outcomes. Recent advances in SRH care, particularly in digital health and community-based interventions, show promise in improving access to culturally appropriate SRH services and information. Co-designing SRH programs with families and young immigrants to adequately acknowledge the unique cultural norms and barriers in SRH is essential in ensuring a high outreach of interventions. Shifts in traditional health policies are needed to ensure that immigrant and minority adolescents are not overlooked and that SRH programs incorporate culturally relevant content that is easily and widely accessible. Despite positive shifts, several barriers remain: limited disaggregated data on diverse populations, inadequate policy attention, and the insufficient scalability and funding of promising interventions. Future research and promotional efforts must prioritise the co-creation of SRH interventions with stakeholders and affected communities, ensuring that services are sustainable, culturally appropriate, and accessible to all adolescents.

## 1. Introduction

The rates of global migration have been continually increasing over the last decade, with almost 4% of the population recorded as immigrants in 2024 [1]. In 2020, immigrants aged 15 to 24 years accounted for 11.3% of the total immigrant population, with this age group accounting for 2.6% of the global population [2]. Adolescence is a pivotal transition period with significant emotional, psychological, and physical changes, making adolescents particularly vulnerable to poor mental and physical health. Furthermore, this life period is important for sexual maturation and exploration, during which adolescents reach reproductive maturity and begin to experiment with romantic and sexual relationships. However, for many adolescents, these important years are disrupted by the, often traumatic, challenges of migration that limit the stable and supportive environment needed to fully explore their development into adulthood [3].

The global rates of SRH service usage are lower amongst immigrants when compared to non-immigrants [4], with a number of structural and individual barriers, such as high costs, cultural beliefs, and language differences inhibiting service access [5,6]. Further, studies have shown that adolescents are significantly less likely than older individuals to access sexual health services, particularly for sexually transmitted infections (STIs) [7], often due to shame, anxiety, and negative stigma [8,9]. Additionally, immigrants have been seen to have higher rates of STIs as compared to non-immigrants [10,11]. Adolescents are likely to carry a higher proportion of these infections due to their biologically immature reproductive systems and increased likelihood of engaging in risky sexual behaviours [12].

Studies have previously shown strong relationships between positive sexual development and overall positive health and wellbeing outcomes [13,14]; thus, it is vital to promote positive adolescent SRH among those most vulnerable, including immigrant and minority populations. Despite the high rates of poor SRH outcomes amongst our immigrant youth populations, strides have been made in recent years to promote adolescent SRH amongst young immigrants. Collaborations between healthcare providers, educators, community leaders, and young immigrants have proven to be a significant driver of change in successfully implementing healthcare provisions [15].

With the rates of global migration ever increasing [1], it is important that we understand the ways in which our health care systems and policies support immigrant and minority youth to access SRH services. This knowledge and understanding will allow us to better tailor services to these vulnerable individuals, thus advocating for greater health and migration outcomes. Therefore, this paper will aim to provide a comprehensive synthesis of the current landscape, challenges, and innovations in adolescent SRH within immigrant and minority communities. We aim to highlight as follows:

- Key challenges to SRH access, including cultural and systemic challenges;
- Recent advances in community-based, school-based, and digital health interventions;
- Policy and advocacy efforts shaping adolescent SRH outcomes;
- Gaps in research and future directions for improving services and access.

## 2. Intersectionality: A Critical Lens for Understanding Immigrant Adolescents’ SRH

**Figure 1:**
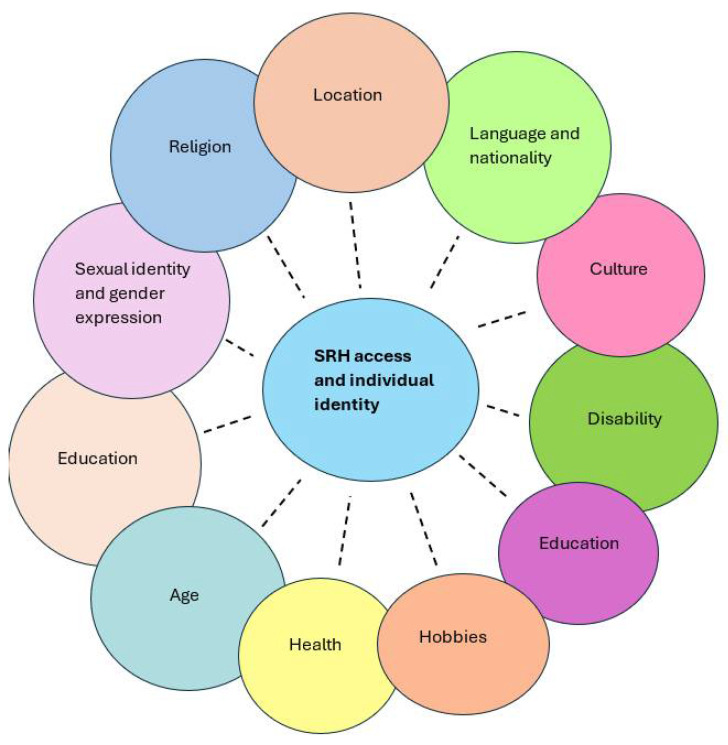
The key dimensions of intersectionality influencing an individual’s identity, beliefs, and access to SRH.

It is well documented that immigrants and minority populations face poorer SRH outcomes in comparison to their non-immigrant counterparts [10,11,16]. This can often be explained through the intersectional barriers they face in accessing health services and information, especially related to SRH, in their new country, including their education, cultural identity, family barriers, and language [6,17,18,19]. Intersectionality, an idea first published by Crenshaw in 1989 [20], can assist in identifying unique and specific inequalities between different groups of people in healthcare and research settings [20,21]. The framework of intersectionality argues that inequalities and injustices faced by groups of individuals are fluid depending on experiences and recognises that all aspects of marginalisation are relevant when identifying why there are inequalities between two groups [22]. The framework shows that prejudice, oppression, and privilege are based upon an incredible array of far-reaching individual, cultural, and societal factors, as seen in Figure 1. The framework depicts that the interconnected experiences and parts of a person form the basis for their opportunities in society.

Whilst young immigrants and minority groups share similar social disadvantages to older immigrants, the interconnection between their evolving biological, social, and cultural identities creates additional barriers and disadvantages when accessing health care [19,23]. Some cultural norms even exclude young men and women who belong in the LGBTIQ+ community, thus disenabling some young people from exploring their sexual identities and accessing education and support regarding sex and sexuality [24,25]. Additionally, many SRH issues faced by young immigrants remain unrecognised, as many cultural norms prevent open discussion with family members or doctors [26,27]. Global structural barriers exist to disadvantage immigrant and minority groups, neglecting their unique and important health care needs and putting them at risk of poor health outcomes [28,29]. For example, many immigrants around the world are ineligible for certain health care services due to their visa status, commonly including SRH services that are then only available to them if independently sought out at a high cost [28]. Further, young immigrants often have missed essential vaccinations and screening for preventable illnesses, placing them at an increased risk of preventable diseases including HPV [30,31]. Previous studies have shown that many structural barriers exist to oppress immigrants and minority groups from accessing even basic primary healthcare, including unclear and complex access pathways [32], a fear and mistrust of services [33], visa status [28], high costs [34], and a low cultural responsiveness of providers [35]. These barriers are further exacerbated in the face of SRH care services, as they are significantly less accessible and face a higher level of stigmatisation in many cultures and communities [36]. This combination of systemic and cultural challenges contributes to the disproportionately high rates of adverse SRH outcomes amongst young immigrants, including higher rates of sexually transmitted infections and unwanted pregnancies [10,37].

It is pivotal for young people to question, explore, and develop their connection to their sexual and gender identity and expression [37]. Young immigrants and minority groups are too often neglected during these pivotal years, inhibiting their personal development and growth, thus placing them at a higher risk of poor SRH outcomes [38]. It is, therefore, vital that we continue to promote positive SRH care and outcomes for young immigrants and minority groups globally through programs that address multiple areas of intersectionality and minimise social and structural challenges.

## 3. Challenges Faced by Adolescents from Immigrant and Minority Backgrounds

### 3.1. Systemic Challenges (Limited Healthcare Access and Policy Barriers)

As seen in Figure 1, the intersectional interplay between minority adolescents’ location, cultural identity, and nationality, as influenced by systemic inequalities, creates substantial barriers to accessing SRH services for adolescents from immigrant and minority communities [39]. Despite the availability of well-funded and technologically advanced healthcare systems, even those in high-income countries, such as the United States (USA) and Australia, face substantial challenges when accessing SRH services. Many European countries, as well as the US, restrict access to national healthcare services to asylum seekers and other immigrants, limiting their abilities to seek curative and preventative care during the early stages of migration [40,41]. Furthermore, many asylum seekers in Europe have reported eligibility to fewer resources to stabilise and improve their health and wellbeing upon migration in comparison to refugees and labour immigrants [42]. Many immigrant adolescents receive limited or no comprehensive sex education both in their home and migrant countries due to language barriers and cultural or religious beliefs that restrict open discussions of sexual health [28,43,44]. Further, school systems often provide non-inclusive education that does not meet the diverse cultural needs of immigrant adolescents, with many wanting to understand SRH from their own cultural view [44,45,46]. Many teachers and health providers lack the essential training needed in discussing SRH and working with marginalised youth populations [44], further limiting the accurate, sensitive SRH information available to immigrant adolescence. Many education policies around the world do not mandate sexuality education training for schoolteachers, thus leaving them blind on how to cover controversial topics in class, often resulting in sensitive topics being left out of the lessons [47].

In addition to the obstacles adolescents face when accessing SRH services and education, existing policies often fail to address their unique needs and requirements [48]. Adolescents who are on new arrival or temporary visas are frequently excluded from comprehensive healthcare coverage, limiting their access to affordable healthcare and further disincentivising them to seek preventative care [49,50]. Anti-immigration policies in some high-income countries, including Canada and the USA, prevent at-risk communities from accessing timely treatment and support for their SRH, including vital HIV treatment and medicines [50]. Additionally, the often-required proof of residence needed for many health insurance schemes and GP registrations prohibits many undocumented immigrants and those with no fixed address from accessing essential services, regardless of their cost or universal accessibility [51,52]. Recent changes to US immigration policies have resulted in the overturning of former legal protections against immigrants in healthcare facilities, thus creating a fear of deportation in those seeking medical help [53]. Regardless of migration status, many young immigrants and their families are left unaware of the SRH resources available to them due to unclear policies around their healthcare eligibility [46]. The low promotion of free SRH resources to immigrant communities and the difficult pathways to finding this information, especially for those with low health and English literacy, further hinders their abilities to source reliable and culturally appropriate information [44,46].

The exclusionary nature of existing policies worldwide exacerbates inequities in SRH outcomes and plays a key role in the overall health and wellbeing of immigrants [54]. Considering the intersectionality of migration status, language, and socio-economic factors is essential in addressing health inequalities amongst immigrant and minority adolescent populations.

### 3.2. Individual Barriers (Cultural Barriers and Stigma)

In addition to systemic challenges, adolescent immigrants and minority groups face incredible individual and cultural barriers to accessing appropriate SRH care after migration. Deeply ingrained beliefs, often carried from their countries of origin and enforced by community members, continue to shape their attitudes towards their SRH, frequently hindering their access to health services [55]. For instance, many Middle Eastern, African and Asian communities see discussions around sexuality, particularly premarital sexual activity, as taboo and are heavily stigmatised and discouraged [45,56,57]. A recent Australian study reported that many young immigrants from Northern and Eastern African backgrounds felt shame and guilt in seeking SRH advice from service providers who were often not compatible with their cultural beliefs and SRH concerns [28]. Further, a recent study found that young South Asian women in the UK often delayed accessing reproductive health services out of a fear of family disapproval, with some further mentioning that they concealed all SRH care access from family members [58]. Research consistently highlights that cultural and religious norms inherited from the countries of origin contribute to a culture of silence and stigma surrounding sexuality. These taboos often persist in host countries, creating substantial barriers to SRH knowledge, education, and access for adolescent immigrants [59]. Addressing these challenges necessitates culturally sensitive interventions, communication, and inclusive health policies that acknowledge, respect, and respond to the diverse cultural and religious values of immigrant communities [60].

The prevailing culture of silence, reinforced by entrenched taboos, extends beyond merely hindering open conversations, but also fosters the proliferation of misinformation and a widespread lack of awareness regarding sexual and reproductive health (SRH) among adolescents from immigrant and minority backgrounds [61]. Evidence from a recent Australian systematic review indicated that young immigrants’ information-seeking behaviours were heavily influenced by their family, friends, and community [62]. As a result, many young immigrants turned to informal sources such as peers or social media platforms for information [38,45]. Many young immigrants further reported that the SRH education received in school systems was not socially or culturally inclusive or relevant [45]. Additionally, studies have shown that many young immigrants hold inaccurate SRH beliefs, especially in relation to contraception and STI transmission [63]. Many young immigrants reported limited access to SRH information and conversations within their family and community networks, and only receiving SRH education in schooling systems [64]. Findings from a South African study indicated that young female refugees had a particularly low knowledge and understanding of reproduction, including menstruation and contraception [65]. This was largely attributed to the lack of structured and in-depth SRH education for women of all ages [65]. This knowledge gap is not solely a result of limited educational access but is influenced by the often enduring cultural narratives sustained by young adults, parents, and communities that associate SRH education with moral transgression or the promotion of promiscuity [66]. Effectively addressing this issue requires more than the mere dissemination of SRH information within communities and schools. It calls for the development and implementation of culturally sensitive, adolescent-centred educational strategies that build trust, challenge taboo, and empower young people to make informed, autonomous decisions about their sexuality and SRH.

Many intersectional barriers, including those in policy, language, and health literacy, greatly hinder young immigrants from accessing essential SRH care and information in their host country, placing them at a greater health disadvantage compared to non-immigrant youth. Continued research and advances in SRH promotion and programs is essential in overcoming these barriers and ensuring that culturally relevant and safe SRH education is provided for all minority adolescents.

## 4. Advances in SRH Promotion for Immigrant and Minority Adolescents

### 4.1. Innovative Interventions

The innovative interventions of recent years, such as digital health programs and community-based interventions, offer promising avenues for improving access to SRH services for young immigrants and minority communities [28,67]. They can be used as tools to overcome the intersecting barriers that often render mainstream healthcare systems inaccessible or mistrusted, and are often able to better address the complex, culturally specific needs of immigrant adolescents and minority communities.

#### 4.1.1. Digital Health

Digital health has emerged as a powerful mechanism for engaging immigrant youth in SRH services. The adaptability, high accessibility, and scalability of this tool render it vital beyond just enhancing access to care, but also to empower adolescents to make informed decisions about their own health [68]. In the United Kingdom, the Brook Digital Clinic offers confidential consultations, contraceptive support, and STI guidance through an online platform with multilingual accessibility tailored to diverse communities [69]. Similarly, Australia’s government-funded 1800MyOptions initiative provides counselling via phone, web, and in-person platforms, focusing on contraception and abortion care for women from immigrant and culturally diverse backgrounds [70]. These platforms help overcome stigma, limited mobility, and language barriers by offering discreet, user-friendly access to evidence-based information and support. A scoping review of digital health interventions for immigrants highlighted their powerful use in delivering sensitive health-related content, including pregnancy and mental health, to immigrants, with many included studies seeing an increase in positive health behaviours and increased health literacy [71]. However, it is also integral that these programs are co-designed with young immigrants to ensure successful acceptance and availability of their use [71,72]. Priority research exercises and research-led focus groups are effective ways to engage adolescent immigrants in all stages of digital health development, ensuring their voices are heard and represented throughout the process [73].

Digital toolkits for information on SRH topics have proven beneficial for diverse youth around the world, allowing them to take agency in their own SRH knowledge and beliefs and obtain accurate and in-depth knowledge in an easily digestible manner [74]. However, digital interventions assume a level of digital literacy and accessibility, potentially excluding an entire group of young immigrants living in low-income or remote areas with little digital accessibility [75]. Overcoming low digital health literacy with the use of images, videos, and multiple language availability has previously proven effective

Digital health platforms can be incredibly useful tools for delivering SRH information to a young population who often look to the internet and their peers for health information; however, it is vital that this information is continually adapting to the changing needs of the audience [74]. Recent systematic reviews have indicated that co-creating digital health interventions with communities and ensuring that members of their community were a part of the research team is important in active community engagement [76]. Engaging community members with the design of digital interventions assists in ensuring their own culture is accurately represented and assists in overcoming the fears and mistrust of online information [75]. This indicates that community-based interventions should be at the forefront of all SRH promotion research and innovations.

#### 4.1.2. Community-Based Interventions

Community-based interventions have also gained traction in increasing access to SRH care amongst young immigrants and diverse communities [77]. For example, a recent Canadian study aimed to co-design a mHealth (online health) program for Canadian youth from immigrant and refugee backgrounds [73]. The program utilised focus groups and community-led engagement to work with young people from immigrant and diverse communities to create a culturally appropriate and easily accessible mHealth platform for SRH information. In Australia, the ‘Health in My Language’ initiative, led by the Multicultural Centre for Women’s Health, employs trained bilingual health educators to deliver SRH sessions to newly arrived immigrant and refugee women in their native languages [78]. Meanwhile, in the Netherlands, the ‘Long Live Love’ programme offers a comprehensive, school-based SRH curriculum tailored for students from diverse backgrounds, integrating cultural sensitivity with education on gender equality and personal agency [79,80]. Similar pilot programmes in Sweden and Germany use trauma-informed, culturally adapted curricula to include refugee youth in school-based health education [81]. A recent scoping review investigating SRH programs for young immigrants found that community engagement, including peer educators and group learning, is integral in improving program engagement and uptake to ultimately combat poor SRH in young immigrants [77]. Additionally, equipping young immigrants with skills and knowledge in research, ethics, and mentorship is a powerful tool to promote active engagement, allowing them to co-lead intervention design and dissemination.

Due to strict cultural practices and limited community-based sex education in communities worldwide, it is important to provide culturally diverse young adults and adolescents with comprehensive sexual education in school institutions. A recent study showed that many young immigrants in Australia felt that the sex education they received after migration did not fully meet their needs. Young Vietnamese women noted that more information directly related to the pressures and cultural issues with sex and sexuality was needed [45]. A pilot study in the USA has shown promising results in engaging young adults from immigrant and refugee backgrounds with SRH education, with the use of a choose-your-own-adventure story [82]. The pilot saw a positive response in increasing young immigrants’ understanding of their SRH, with participants also urging the need for greater diversity in scenarios, especially in relation to consent awareness [82]. The EveryBODY Education Program in Victoria, Australia, is an in-school education program that provides SRH education to immigrant and refugee communities free of charge, covering a wide range of SRH topics including LGBTQIA+ information [83,84]. Educators of this program have noted their emphasis on the importance of reminding young immigrants that the programs do not override any values learnt at home or in their community but are instead presented with information to consider alongside their established values and beliefs. Reiterating this idea, for both students and their families, encourages the students to actively engage in all aspects of the education session and curiously ask questions of the trained educators [83]. Education policies in Quebec are strongly aimed at collaboration between school settings and immigrant families, encouraging immigrant parents to engage with their children’s education and learning [85]. This complementary education can see positive impacts in developing communication and knowledge sharing between immigrant children and parents [85]. However, this mode of education must be built on strong communication between immigrant communities and schools in order to ensure that culturally appropriate and safe information is being taught and discussed in classrooms. Fostering environments filled with empathy, support of self-efficacy, reflective listening, and understanding are integral in connecting families with SRH programs. Opening spaces for curiosity, safe communication, and respect for cultural ideas are also essential for effective community-based interventions [86]. Cultural taboos must be sensitively addressed and discussed with both young adults and their families, improving knowledge, confidence, and engagement in programs with all family members [87].

### 4.2. Policy Shift

To sustain and amplify the impact of digital and community-based interventions in SRH, a parallel and credible shift in policy frameworks is essential. Whilst traditional health policies frequently overlook the immense challenges faced by immigrant adolescents and minority communities, a growing number of governments and public health institutions are adopting more inclusive, equity-oriented approaches [37,88]. For example, New Zealand has introduced youth-specific health equity policies that allocate dedicated funding for SRH services accessible to immigrant youth [89]. This approach promotes the inclusion of culturally relevant content in public education curricula, thereby reinforcing community-led SRH programmes and signalling a broader move towards structural transformation [89]. Such policy developments not only acknowledge and validate the diverse lived experiences of immigrant youth but also lay the foundation for sustainable, system-level change [90,91]. The South Australia Youth Action Plan (2024–2027) urges the need for greater access to education and early support with sexual and reproductive health, especially for those from culturally and linguistically diverse backgrounds [92]. The plan works with the Australian Government to deliver equitable access to resources and opportunities for all young people, thus urging the government to improve SRH resources to young immigrant communities [92]. Many countries, including Australia and the US, require domestic SRH screening guides for refugees upon entrance to their country [93,94]. The Australian Medical Association hold strong policies for doctors who treat refugees and humanitarian entrants, including those who require an initial health screen. Policies and guidelines are in place for Australian doctors performing these tests to ensure that all new arrivals are given compassionate and culturally sensitive health care, which is particularly important to young immigrants [93].

Ultimately, bridging the gap between policy and practice requires governments to institutionalise inclusivity, ensure the equitable distribution of resources, and meaningfully engage affected communities in shaping SRH agendas [90,91].

## 5. Gaps and Future Directions–Research Needs and Policy Recommendations

Despite notable progress in expanding adolescent SRH services through digital platforms, community-led models, and policy innovations, several critical gaps persist that hinder equitable access and long-term impact for immigrant and minority communities.

1. A Lack of Disaggregated Data

A fundamental limitation is the persistent absence of disaggregated data by migration status, ethnicity, age, gender identity, and other relevant social determinants [95]. Most national health surveys and administrative datasets do not systematically capture these variables, resulting in a limited understanding of the unique SRH needs of diverse adolescent populations. Without detailed data, it is difficult to identify trends, monitor disparities, or evaluate the effectiveness of targeted interventions [96]. This lack of visibility contributes to the continued marginalisation of immigrant and minority adolescents in public health planning and policy development and continues to negatively impact the outcomes of targeted health interventions. Furthermore, where disaggregated data do exist, they are often inconsistent, outdated, or collected using non-standardised methodologies that preclude cross-country comparisons or longitudinal tracking [95]. The absence of high-quality, representative data also limits the ability to conduct robust, equity-focused research and weakens the evidence base for culturally appropriate policy responses.

2. Limited Scalability and Integration of Culturally Adapted Interventions

Although numerous community-based and culturally sensitive SRH initiatives have shown promise in local contexts, their reach and long-term sustainability remain constrained. Many of these programmes are short-term pilots, supported by time-limited funding or led by non-governmental organisations without institutional backing [97,98]. As a result, successful interventions often remain fragmented and fail to scale beyond isolated communities or regions. Further, due to the lack of specific research and data on young immigrants and diverse communities, it is difficult to accurately understand what the greatest needs and wants are for these populations [99]. Co-creating research and interventions that strengthen the connections between key SRH stakeholders and immigrant and diverse communities are essential in developing culturally appropriate health-based programs that are useful and sustainable to the intended audience [100]. A further challenge is the integration of these programmes into mainstream healthcare and educational systems. Despite their proven effectiveness, culturally adapted interventions frequently operate outside formal service delivery structures, limiting their uptake, continuity, and policy relevance [77,100].

Research shows that transportation and a lack of time are major barriers to many immigrant and diverse communities, especially for those with physical disabilities [101], to accessing health care services [102]. If these specifically adapted interventions are not available to young immigrants and diverse communities through more formal, structured delivery structures that cater to these barriers and needs, uptake and continuity will remain low. Further, institutional resistance and a lack of political will can hinder the mainstreaming of culturally tailored models [103]. There is also a need for mechanisms to evaluate and adapt these health programs across diverse settings to ensure that cultural relevance is preserved in the ever-changing world.

3. Inadequate Policy Attention to Structural and Intersectional Inequities

Current health policies in high-income countries frequently overlook the intersectional nature of the barriers faced by immigrant and minority adolescents. While some policies recognise broad categories such as “immigrant” or “ethnic minority,” they often fail to address how overlapping factors—such as legal status, language proficiency, socio-economic disadvantage, trauma history, and gender—compound vulnerability and shape access to SRH services [104]. This limited policy scope results in fragmented service provision, where adolescents may encounter different levels of access depending on their visa category, region of residence, or service provider [105]. Moreover, policies often assume uniformity within minority populations and neglect the internal diversity of experiences, cultural norms, and health beliefs. Without comprehensive, intersectional policy frameworks, efforts to improve SRH outcomes will remain superficial and be unable to address the root causes of inequity [28].

4. Insufficient Youth Engagement in Programme Design and Evaluation

Another major gap is the lack of meaningful participation from immigrant and minority adolescents in the design, delivery, and evaluation of SRH programmes [106]. Existing models often treat adolescents as passive recipients of care rather than as active stakeholders with valuable insights and agency. This top-down approach limits the relevance, acceptability, and effectiveness of interventions, particularly among groups whose voices are rarely included in health decision-making processes. Participatory models that involve adolescents in shaping programmes from needs assessments to content development and service delivery are still the exception rather than the norm. Promoting youth engagement mechanisms across health systems, educational settings, and community platforms is vital to ensuring programs align with young immigrants lived experiences, in turn strengthening trust and enhancing the uptake of services [107].

5. Limited Evidence on the Long-Term Impact of Digital SRH Interventions

While digital health tools have rapidly gained popularity as an accessible and scalable solution for delivering SRH information and services, evidence on their long-term effectiveness remains sparse. Most existing evaluations focus on short-term indicators, such as knowledge gains or platform usage, with little attention to sustained behavioural change, clinical outcomes, or the reduction in health disparities over time [108]. Moreover, digital platforms often assume universal access to technology and digital literacy skills, which is not the case for many immigrant adolescents. Barriers such as limited internet connectivity, low digital literacy, and concerns over privacy or data security disproportionately affect youth from disadvantaged or recently arrived communities [109]. These factors may exacerbate existing inequalities rather than resolve them, particularly in underserved or rural areas [110]. Moreover, there is a lack of culturally nuanced evaluations into digital tools that are often created for a universal audience. Interventions developed in dominant cultural contexts may inadvertently reinforce exclusion or fail to engage minority and diverse youth communities. Future research, such as that being conducted in the USA by Dear Digital Equity [111], must explore how digital platforms can be adapted to meet diverse cultural, linguistic, and technological needs while maintaining clinical accuracy and user engagement [112].

6. Fragmented Funding and Inconsistent Evaluation Frameworks

Sustainable financing remains a major challenge for SRH initiatives targeting immigrant and minority adolescents. Funding streams are often project-based, unpredictable, and not aligned with the long-term strategic goals of the programs. As a result, effective interventions may face discontinuation once initial grants expire, and promising programs may never gain the traction needed to thrive. This funding instability undermines efforts to build institutional capacity, retain trained personnel, and ensure consistent service delivery [113]. Compounding this issue is the lack of standardised evaluation frameworks that can be used to assess the effectiveness and equity of SRH interventions across diverse settings. Many programmes are evaluated using ad hoc or non-comparable metrics, limiting the transferability of lessons learned. There is a pressing need for coordinated evaluation systems that incorporate qualitative and quantitative indicators, track progress over time, and include measures of cultural responsiveness, youth satisfaction, and structural impact [114].

## 6. Future Directions

To address these multifaceted gaps, future efforts in research and policy should prioritise the development of co-created, inclusive data systems that capture the full diversity of adolescent populations. Health systems must invest in longitudinal research, integrate culturally adapted interventions into standard care, and restructure policies to reflect the intersecting determinants of SRH inequity.

Additionally, youth engagement should be prioritised and formalised through participatory governance structures and funding mechanisms that support adolescent-led initiatives. Digital platforms must be developed and assessed with attention to cultural relevance, digital equity, and long-term outcomes. The co-designing of all interventions with multiple community members is essential in creating widespread and trusted programs. Future research needs to focus on understanding the specific needs of adolescent immigrants, allowing them to voice their priorities in SRH information and delivery. Community engagement, with adolescents, family members, and educators, must be at the forefront of future research, ensuring all key stakeholders are considered in design and implementation processes.

Finally, sustainable financing models and harmonised evaluation standards are essential to ensure that progress is measurable, replicable, and resilient over time. Together, these strategies can build a more equitable, responsive, and sustainable SRH landscape that meets the needs of all adolescents, regardless of their migration status, cultural background, or socio-economic position.
